# Effect of mindfulness on online impulse buying: Moderated mediation model of problematic internet use and emotional intelligence

**DOI:** 10.3389/fpsyg.2022.1012331

**Published:** 2022-12-06

**Authors:** Nitin Simha Vihari, Nishit Kumar Sinha, Akansha Tyagi, Saurabh Mittal

**Affiliations:** ^1^Middlesex University Dubai, Dubai, United Arab Emirates; ^2^Indian Institute of Management Indore, Indore, Madhya Pradesh, India; ^3^Chitkara Business School, Chitkara University, Punjab, India; ^4^Indian Institute of Management Lucknow, Lucknow, Uttar Pradesh, India

**Keywords:** mindfulness, access to the internet, problematic internet use, business, online impulsive buying behavior, developing country, mental health, emotional intelligence

## Abstract

**Introduction:**

Online impulse buying behavior is an unplanned urge to buy a product or service in an online setting and it has several negative consequences for customers, such as guilt and financial distress, and e-commerce firms, such as higher returns and customer complaints. Evidently, it is important to examine the various psychological processes which may assist in a better understanding, therefore addressing the high prevalence of online impulse buying. This study builds upon self-regulation theory to explore how mindfulness influences online impulse buying, and examines problematic internet use as a mediator in the relationship between mindfulness and online impulse buying. Further, this study investigates how emotional intelligence as a moderator plays the role of a suppressant on the adverse impact of problematic Internet use which fuels online impulse buying.

**Method:**

A total of 598 individuals working with various servicebased industries responded to the questionnaire. Multiple regression and moderated mediation analysis was used using SPSS and AMOS for analyzing the data.

**Result:**

Problematic internet use mediates the relationship between mindfulness and online impulse buying behavior. Emotional intelligence negatively moderates the relationship between problematic internet use and online impulse buying behavior.

**Discussion:**

This study findings outlined the inverse relationship of mindfulness & online impulse buying, along with the mediating effect of problematic internet use between mindfulness and online impulse buying. Further, this study showed how emotional intelligence played an important role as a moderator by suppressing the adverse impact of problematic Internet use and preventing online impulse buying. The study offers implications to online marketers in regulating the unplanned purchase process—while minimizing uninhibited buying behavior that leads to regret, and the subsequent intention to return products. Further, social and theoretical implications are discussed.

## Introduction

Impulsive buying is sudden and unplanned purchase often made under the influence of stimulus and is frequently associated with a “powerful and persistent urge to buy something immediately” (Rook, 1987, p.191), driven by emotional motivation and hedonic goals (Liao et al., 2009). Research on impulse buying has evolved from ‘what (product)’, ‘where (retail store environment and its elements)’, ‘who (categorizing individuals as impulsive buyers or not)’ to exploring ‘when’ and ‘why’ such behavior exists (Vohs and Faber, 2007; Mandolfo and Lamberti, 2021). Since impulse purchase often leads to negative consequences for customers such as, post-purchase negative emotions like guilt (Rook, 1987), a vicious trap of financial hardships (Fenton-O'Creevy et al., 2018) or diminished financial well-being (Nanda and Banerjee, 2021) and for business firms such as, higher intention to return goods and customer complaints (Zeelenberg et al., 2000), a need is felt to recognize and better understand the ‘why’s or the drivers of such behaviors (Frigerio et al., 2020; Thürmer et al., 2020; Özyörük, 2022). Extant literature suggests that antecedents of impulsive buying can be distinguished into four broad categories of variables: *dispositional*, *situational*, *sociodemographic*, and *dispositional* or *situational interaction* (Amos et al., 2014; Rodrigues et al., 2021) and that gaps exist in understanding various dispositional or internal processes which drive impulse buying (Badgaiyan and Verma, 2014; Huang, 2016; Dhandra, 2020).

With the growing prevalence of e-commerce and social commerce among consumers, the occurrence of online impulse buying has drawn significant research interest (Zhao et al., 2021). Fuelled by the easy access to the Internet, ease of search for information and purchase (Sağkaya Güngör and Ozansoy Çadırcı, 2022) through multiple online shopping platforms, and effortless delivery, online impulse buying is a growing phenomenon (Jeffrey and Hodge, 2007; Verhagen and van Dolen, 2011). Some researchers highlight that impulse buying contributes to more than 50% of online shopping (Kimiagari and Asadi Malafe, 2021). Unfortunately, the Internet has provided the two commonly associated situational antecedents of impulsive buying: proximity and mood (Vohs and Faber, 2007), with the just-right germinating environment, which has further fuelled online impulsive buying. With this background, researchers have underscored the need to examine the customers’ internal characteristics that may shed light on customers’ impulse online buying behavior (e.g., Chan et al., 2017; Guendelman et al., 2017). In this regard, mindfulness has been considered as an antidote to online impulse buying (e.g., Gao et al., 2021). Mindfulness is associated with reduced impulsivity, higher emotional stability, higher self-esteem, and lesser urge to act on consumerist messages which promote impulsive buying (Brown and Ryan, 2003; Peters et al., 2011; Papies et al., 2012, 2015). While the role of mindfulness has been examined in reducing impulsive buying (Dhandra, 2020), a gap exists in exploring this relationship while accounting for customers’ internal characteristics that are reflective of their online behaviors (Shonin et al., 2013).

One less-discussed aspect of customers’ internal characteristics, which is of utmost relevance while understanding online impulse buying is problematic Internet use (PIU). PIU is termed as dysfunctional use of the Internet (Caplan, 2010) and is often associated with “difficulties with behavioral impulse control, ultimately resulting in negative outcomes associated with their [individual’s] Internet use” (Caplan, 2010, p. 1090). Behavioral scientists explored this domain from the lens of self-regulation theory to find that impulsive behavior results from a lack of control over our thoughts, emotions, and actions (Baumeister, 2002). Importantly, the COVID-19 pandemic has further fuelled PIU (Király et al., 2020), making the case to examine the role of PIU concerning impulsive online buying even more relevant.

Further, extant research indicates that being aware of our emotions and thoughts might help prevent any impulsive behavior, including excessive time spent on the Internet (Vohs and Faber, 2007). “The ability of a person to regulate his or her emotions, enabling a more rapid recovery from psychological distress,” known as Emotional Intelligence (Law et al., 2004, p. 484), is an important concept that has proved to be an effective remedy for impulsive buying behavior. Also, mindfulness promotes better emotion regulation abilities and is negatively related to adverse affective experiences such as depression (Mesmer-Magnus et al., 2017) and loneliness (Jin et al., 2020), which are some of the distal drivers of PIU (Davis, 2001). Multiple studies suggest that comprehending customers’ internal characteristics are paramount to develop a sound understanding of impulsive online buying (Chan et al., 2017; Iyer et al., 2020; Kimiagari and Asadi Malafe, 2021).

In this study we investigated the moderating role of EI in the relationship between PIU and online impulsive buying behavior for the following reasons. First, EI is considered an important attribute which may act as a buffer when people are confronted with risk factors or Internet addictions (Peng et al., 2019). Second, literature supports the moderating nature of EI in combating the addictive behavior (Peter and Honea, 2017). Third, individuals with high level of EI can regulate their emotions more effectively than the individuals with lower level of EI and are less impulsive in making decisions. They are less likely to fall prey to external sources such as the internet to alleviate negative emotions (Khoshakhlagh and Faramarzi, 2012) which prevents the users from indulging into internet addiction.

Based on the above discussion, the objective of this study is threefold. First, the study examines the relationship between mindfulness and impulse online buying behavior. In doing so, we attempt to reaffirm the beneficial role of mindfulness, a malleable trait, in promoting individual well-being (Bahl et al., 2016). Second, this study analyses the association between mindfulness and impulse buying in the online realm through a previously unidentified mediating mechanism in the form of PIU. Examining the influence of PIU in the mindfulness and online impulse buying behavior association may help us better comprehend the processes through which mindfulness may reduce impulsive online buying. Third, we explore EI as a suppressant on the negative influence of PIU in promoting online impulse buying. A better and nuanced understanding of the interplay between these psychological constructs presented by this study may assist in the formulation of more refined and focused interventions that make customers aware of their impulsive buying behavior, and promote consumer well-being.

As Iyer et al. (2020) pointed out that marketers may develop “stimuli that both facilitate unplanned purchases and discourage purely uninhibited impulsive purchases that may lead to later regret and consumer dissatisfaction” (p. 400), the online marketers may devise external interventions to stimulate the informed decision-making process by the customers. Though such interventions may discourage immediate sale, which potentially leads to customer dissatisfaction, customers may extend future patronage to such online marketers. The present study offers few ways which may prove effective in augmenting customers’ awareness level to discourage sudden and unplanned online impulsive behavior.

Behavioral scientists explored impulsive buying behavior domain from the lens of self-regulation theory and found that impulsive behavior is a consequence of lack of control over our thoughts, emotions, and actions (Baumeister, 2002; Iyer et al., 2020) and are not able to postpone their instant gratification (Silvera et al., 2008). People find solace in spending time on internet as it gives them instant gratification, be it the social media impressions or online shopping (Gjoneska et al., 2022). Research indicates that being aware of our emotions and thoughts might help in preventing from excessive internet time or any kind of impulsive behavior (Vohs and Faber, 2007). In the light of the discussion, the study aims to understand the interaction of consumer behavior with consumer psychology and their characteristics, that how mindfulness relates negatively with online IBB through the mediating mechanism of PIU, and moderating effect of EI.

## Theoretical framework and hypotheses formulation

Self-regulation theory (Baumeister, 2002) suggests that failure of self-regulation process would lead to addictive and impulsive actions. To explain the various psychological mechanisms involved in the self-regulation process, Baumeister (2002) considered the role of the executive function. Executive function includes the ability to monitor and update the various contents of attention, and consequent flexibility to move around within appraisals or mindsets (Miyake and Friedman, 2012). Self-regulation has been explained as an instance when individuals put efforts into monitoring and modifying their thoughts and behaviors to achieve the desired purpose (Baumeister et al., 2007). In contrast, failure of self-regulation has been defined as any disruption in this procedure.

Internet habits are augmented by automated and unconscious processing of the environmental cues obtained through recurrent and long Internet sessions (LaRose, 2010). In the absence of self-regulation, individuals fail to observe their dysfunctional Internet consumption, reducing awareness and capacity to contain undesirable online behavior (LaRose, 2010). Whereas being able to observe our emotions, direct our attention to monitor our thoughts, and awareness of the internal and external stimuli could prevent indulging in various dysfunctional usage of the Internet (Mascia et al., 2020). Especially, in this era of the digital revolution, studies (e.g., Molden et al., 2016) have demonstrated that multiple psychological factors are responsible behind people’s ability to contain the impulsive responses, and shifting between assessment of their internal emotional responses while setting and pursuing their goals. Mindfulness has been cited as one of those factors.

Role of mindfulness in the process of self-regulation (Masicampo and Baumeister, 2007) is well-established. Mindful people possess higher present-moment awareness that promotes thoughtful decision-making process (Karelaia and Reb, 2015) and contains automaticity in behavior (Brown and Ryan, 2003), thus acting as a strong tool to develop the ability of self-control. Similarly, studies show that emotions and impulses, when controlled and regulated in the right direction, can prevent indulging in excessive internet usage and impulsive behaviors (Mascia et al., 2020). The social-cognitive view of self-regulation and emotional intelligence (Martinez-Pons, 2000) also strengthens the framework of our study by highlighting the role of emotional intelligence in regulating our ability to defer gratification by means of impulsive online buying behavior. Thus, we lay the foundation of this study from the lens of self-regulation theory (Baumeister, 2002) which helps us understand the dynamics of the variables chosen and the nuances of the relationship shared among them.

### Mindfulness and online impulse buying behavior

Mindfulness is “the state of being attentive to and aware of what is taking place in the present” (Brown and Ryan, 2003, p. 822). Mindfulness reflects conscious awareness of the present moment occurrences in non-judgmental way (Kabat-Zinn, 1990). Mindful attention and awareness enable greater access to the cognitive process, through which individuals’ reperceive’ mental reactions to various stimuli, thoughts, and emotional experiences as transient (Papies et al., 2012) and merely mental events (Shapiro et al., 2006). Such mindful orientation leads to an undoing of the mechanical processing of the impulses and acts as a deterrent against the ‘auto-pilot mode’ of unconscious automated judgment (Nguyen et al., 2019). The greater focus of mindfulness lies in the way perceptual images are processed by mindful individuals, who are “able to disidentify from the contents of the consciousness (i.e., one’s thoughts)” (Shapiro et al., 2006, p. 377), to realize that these thoughts are not me (Deikman, 1982). Thus, mindfulness allows for more deliberate decisions about one’s choices. Based on this rationale, mindful mechanisms have been explained as the principal agent behind reducing impulsivity, enhanced self-esteem, emotional stability, and reduced responsiveness to market-propagated consumerist messages (Brown and Ryan, 2003; Peters et al., 2011; Papies et al., 2012).

Mindfulness is proposed to have a negative relationship with online impulsive buying behavior for multiple reasons. First, mindful attention involves simply observing and attending to moment-to-moment experiences. Mindful individuals perceive the uniqueness of the moment, independent of its relationship with the immediate earlier or future moment (Nilsson and Kazemi, 2016). Being mindfully aware is also associated with a balanced and deliberative mind (Kabat-Zinn, 1990) that can assess the relevance of the information and cues present in the environment, supporting a thoughtful decision-making process (Karelaia and Reb, 2015); thus, breaking down thinking patterns and the resultant automaticity in one’s behavior.

Second, impulse buying behavior emanating from general impulsivity is characterized by deficient self-regulation (Fenton-O'Creevy et al., 2018). The non-judgmental aspect of mindfulness encourages a non-evaluative attitude toward experiences, thoughts, and feelings through conscious attention toward one’s own mental experiences (Bishop et al., 2004), thus promoting greater self-regulating ability (Evans et al., 2009). Such individuals are able to monitor their impulses and refrain from acting upon the affective experiences caused by internal stimuli (e.g., emotional states, cognitions) and external stimuli (e.g., electronic commerce website content). Being mindfully aware of the stimuli allows for thoughtful response (Peters et al., 2011), making it less likely for them to indulge in impulsive online buying.

Third, IBB literature supports the presence of situational factors, such as extreme affective state (positive or negative affect) as a motivator behind impulse buying (Vohs and Faber, 2007). Mindfulness promotes present-moment awareness in a non-judgmental or accepting way, which helps in enhancing emotion regulation abilities (Brown et al., 2007). People with strong emotion regulation abilities are better equipped to modulate the affective states through multiple strategies, such as avoidance (refusing to experience certain emotions) and without any interference from ruminating thoughts (Gross, 2013). This non-reactive awareness of internal experiences and affective cues helps mindful individuals avoid maladaptive and counterproductive behavior (Baer et al., 2006), including impulsive buying behavior at electronic marketplaces.

*H1*: Mindfulness is negatively associated with online IBB.

### Mindfulness and problematic internet use

Mindfulness is related to fewer behavioral and addictive problems, such as substance use (Fernandez et al., 2010), owing to mindful people’s greater self-regulation of attention (Bishop et al., 2004). In contrast, PIU is identified as a problem of fleeting attention and deficient self-regulation (Kim et al., 2009; Kim and Davis, 2009), characterized by an inadequate self-conscious process to monitor and adjust one’s behavior in the online space (LaRose et al., 2003; LaRose, 2012). Since mindfulness promotes moment-to-moment experiences (Felder et al., 2012), mindful individuals possess a greater ability to break the automatic behavioral processes found to be associated with the inception and spread of addictive behavior (Ostafin and Marlatt, 2008).

Several studies have exhibited the positive association between mindfulness and several well-being indicators, including greater satisfaction with life and lower negative affect (Brown and Ryan, 2003). Individuals satisfied with their lives have a reduced preference for online social relationships and are less likely to engage in addictive online activities (Shen et al., 2013). The core aspects of mindfulness help individuals present themselves in respective social circumstances periodically, with reduced social anxiety (Dekeyser et al., 2008). Contrastingly, Davis (2001) posited that several components, such as social anxiety and depression, act as the necessary distal cause of PIU. Caplan (2002) contended that individuals with these psychosocial issues might prefer online conversations over face-to-face interactions. Following these findings, mindful individuals are expected to have a lower preference for compulsive online interaction over face-to-face interaction and are less likely to resort to web for mood regulation. Thus, we expect mindfulness to be negatively associated with PIU.

*H2*: Mindfulness is negatively associated with problematic Internet use.

### Problematic internet use and online impulse buying behavior

PIU literature supports the idea that people experiencing several negative affective experiences such as anxiety, depression, and fatigue are more inclined to use the Internet in a dysfunctional manner (Oraison et al., 2020). Spending more time on the Internet, such individuals find less time to develop social relationships in real life with inadequate satisfaction with the psychological need for relatedness (Li et al., 2016). Thus, a negative feedback loop is initiated and fuelled. Negative affect leads to increased Internet use, which further causes alienation from social context and face-to-face relationships, steering them toward activities such as online impulse buying, to compensate for the affect imbalance (Wong et al., 2015). Consistent with the argument, Beatty and Ferrell (1998) argued that time spent browsing or buying without specific intent may fulfill the emotional worth of the spent effort, thus bringing greater satisfaction.

A negative mood leads to “a desire to conserve one’s resources” (Moore et al., 1976), compelling people to circumvent the cognitively challenging process of deliberation and rational evaluation of action and consequences. Baumeister et al. (2013) demonstrated that individuals experiencing negative moods might lapse in self-regulation to balance their mood state. To get over the negative mood, individuals might seek an instant reliever (Moore et al., 1976) in the form of impulse buying which is a strategy for mood regulation (Fenton-O'Creevy et al., 2018). Such an explanation is in sync with Impulse Buying theory, which suggests that extreme moods, positive or negative (Flight et al., 2012), may fuel impulse buying.

Carver and Scheier (1998) explained self-regulation mechanism using the system approach. The critical features of the system approach are: the existence of a standard, monitoring the present condition, a comparison between the present and the desired situation, and the probable list of actions as measures of counterbalancing discrepancies. A basic illustration of the system approach is the heating equipments at home. The heating system has the desired temperature (standard), a monitoring instrument (thermostat), and finally, a tool to balance the temperature (heater). The system approach is present in our biological existence and the psychological functioning of human beings in the form of self-regulation. Because individuals have limited resources to regulate their behavior, prolonged exposure to the Internet depletes their self-regulatory resources and may also provide opportunities to become impulsive buyers (Vohs and Faber, 2007). The inclusion of PIU in examining impulsive buying is warranted since withering self-control levels lead to more impulsive buying (e.g., Vohs and Faber, 2007), and extant research suggests that individuals high on PIU suffer from a loss of self-control (Caplan, 2010).

*H3*: Problematic Internet Use is positively related to online impulse buying behavior.

### Mindfulness, problematic internet use, and online impulse buying behavior

The mentioned rationale and literature demonstrate that mindfulness is negatively associated with PIU (e.g., Arslan, 2017) and IBB (Dhandra, 2020). Li et al. (2016) suggest excessive Internet use is a cognitive and psychological diversion technique used by individuals to regulate the negative affective experiences. Overwhelmed by the ‘irrational self’ caused by extreme affective experiences (Hobfoll et al., 2018), buying online impulsively becomes the preferred mood-regulation strategy and source of immediate gratification for such individuals. In contrast, a mindful non-reactive stance fosters a tendency to have a free movement of thoughts and feelings, instead of being absorbed by them (Baer et al., 2008), creating a psychological distance between one’s emotional state and consequent dysfunctional online behavior. Online IBB is a function of external stimuli coupled with consumer’s internal characteristics (Kimiagari and Asadi Malafe, 2021). As highlighted in the past studies, preliminary findings related to a negative association between mindfulness and PIU (e.g., Gámez-Guadix and Calvete, 2016) but, a gap still exists with reference to Indian context in the post-pandemic era. Thus, in the presence of mindfulness, the individual’s dysfunctional propensity to use the Internet is expected to diminish, negatively affecting their online impulsive buying behavior.

*H4*: Problematic Internet use mediates mindfulness and online impulse buying behavior relationship.

### Problematic internet use, online impulse buying behavior and emotional intelligence

Previous studies on buying behavior suggest that individuals indulge in random buying under the influence of extreme emotional experiences. For instance, while angry or under stress, individuals shop to uplift their mood (Parsad et al., 2021). Rook (1987) argued that the impulse to buy might trigger emotional conflict in people’s minds and potentially disrupt customers’ behavior patterns. An impulsive buyer finds emotional comfort in buying spontaneously and is most often a recreational purchaser whose purchase decisions depend heavily on their short-term disposition (Zhang et al., 2022). Côté and Hideg (2011) argued that people can have knowledge of a particular product yet make poor buying decisions if they are not emotionally intelligent and easily fall into the trap of emotional marketing. Individuals low on emotion regulation frequently use impulsive buying as a compensatory mechanism to alleviate, repair or manage their emotions (Kemp and Kopp, 2011).

According to the stress literature, anxious individuals tend to make decisions out of their personal experience rather than using rationality. Moreover, Cartwright and Cooper and Cartwright (1997) explained that individuals who are capable of regulating their emotions are likely to appraise a potential threat and cope up with it in adaptive ways.

**Figure 1:**
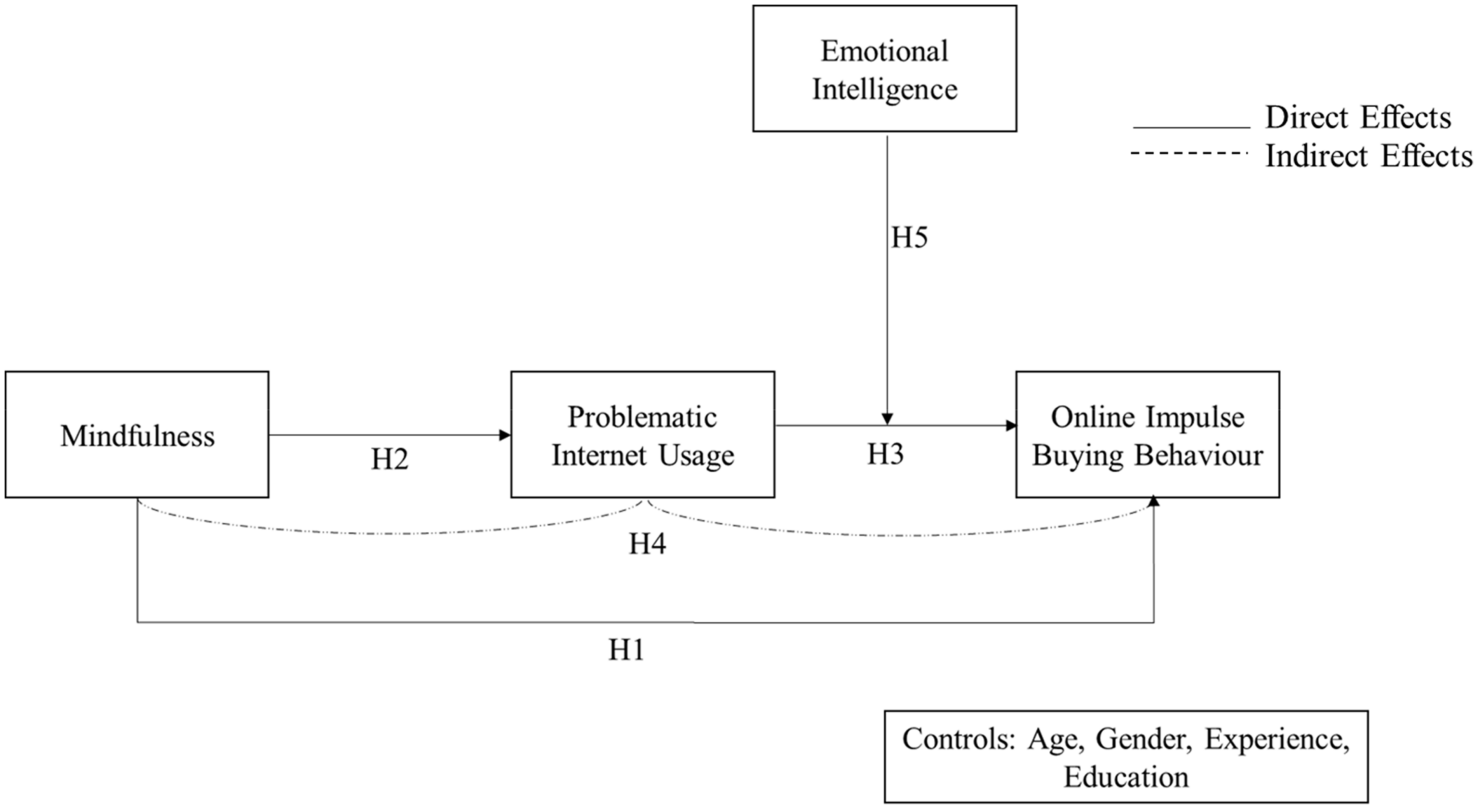
Conceptual Framework of the study.

PIU is associated with mood dysregulation (Caplan, 2010), often leading to high impulsivity (Vohs and Faber, 2007), while EI relates to managing the emotions well and reduces impulsive buying (Peter and Krishnakumar, 2010). Individuals with low EI are more susceptible to developing PIU (Yang et al., 2005). Few studies demonstrated the relationship between PIU and impulsivity (e.g., Dawe et al., 2004). Thus, it seems plausible that EI acts as a buffer to reduce the negative consequences of PIU. More emotionally intelligent individuals are expected to be more equipped to differentiate between various emotions and possess a higher ability to regulate as well as use emotions to direct their thoughts and behavior (Wong and Law, 2002). Such customers may find it easier to avert themselves from becoming absorbed in adverse emotional states which initiates mood repair process, inclusive of online impulsive buying activity. Also, since IBB has an element of emotional response associated with it, and consumer culture indicates to the ‘sense of meaning’ experienced through consumption, it shows that controlling or modifying this element (emotion) of one’s personality (Kazemi and Sorooshnia, 2019) might change the buying behavior of consumers (Vohs and Faber, 2007). Relatedly, Chiou et al. (2005) argued that people with high EI are less likely to succumb to impulse buying desires since they can better manage and interpret their affective responses and use multiple mitigating strategies accordingly. In support, Peter and Krishnakumar (2010) demonstrated the beneficial effect of EI in containing impulse buying (Figure 1).

*H5*: Emotional Intelligence has a moderating effect on online impulse buying behavior, such that emotional intelligence acts as a negative moderator between problematic Internet use and online impulse buying behavior.

## Methodology

### Participants and procedure

Before the actual survey, we have conducted a pre-test to ensure the appropriateness of face validity, formatting, and wording with the help of four senior academicians who has substantial experience in consumer behavior (Jager et al., 2017; Howard, 2018). As the construct measurement scales were adapted from the published studies, experts suggested very few changes such as randomization of items. For the actual survey, the data were collected from 1,064 working professionals from various service based industries across four different cities in India. After obtaining prior approval from the respective organizations, online questionnaires were sent to the respondents. An initial response set of 634 (60.61%) was received, and after checking for missing data and outliers, the final set of 598 responses (57.17%) was used for the analysis. We have used the sample size criteria (N > 50 + 8 m) given by Tabachnick et al. (2007) (‘N’ – sample size and ‘m’ – number of independent variables) and Holter’s Index (minimum 200 questionnaires in SEM) to compute the sample size and 598 responses are well within the acceptable limits. We deployed both the procedural as well as statistical methods to control for method biases, as recommended by Podsakoff et al. (2012). Following steps were taken related to procedural aspects: First, the respondents were made aware of their voluntary participation. Second, to contain the socially desirable responses, the respondents were assured of data analysis in an anonymized manner at the aggregate level only. Third, respondents were informed that there is no right or wrong answer. Fourth, to prevent the probable influence of the study motives, the participants were not made aware of the constructs as well as the conceptual model. Finally, the responses were collected through self-administered survey instead of personal interview method.

Table 1 presents the sample characteristics. Notably, most of the respondents were male (66.39%), which is generally observed in the target respondents.

**Table 1:** Demographic description of the respondents.

**Variable**	**Details**	***N***	**Percentage**
Gender	Male	397	66.39
	Female	201	33.61
Age	Less than 22 Years	61	10.20
	22–30 Years	192	32.11
	31–40 Years	249	41.64
	Above 40 Years	96	16.05
Industrial sectors	IT/ITES	213	35.62
	Banking	156	26.09
	Telecommunications	137	22.91
	Pharmaceuticals	92	15.38
Education	Undergraduate	362	60.54
	Graduate	198	33.11
	Doctorate	38	6.35
Total work experience	0–2 years	204	34.11
	3–8 years	179	29.93
	9–15 years	132	22.07
	More than 15 years	83	13.88

### Questionnaire

There are two sections in the survey questionnaire. Specifically, Section 1 of the survey included questions related to demographics (gender, age, industrial sector, education, and experience), followed by the items related to the four study constructs in Section 2.

#### Mindfulness

The study used 12-items Cognitive and Affective Mindfulness Scale-Revised (CAMS-R) developed by Feldman et al. (2006) to capture mindfulness. This scale represents various dimensions of mindfulness such as attention, awareness, non-judgment, and present-focus (e.g., “I can accept things I cannot change”). Respondents expressed their (dis) agreement on a Likert type four-point scale extending from 1 (“rarely/not at all”) to 4 (“almost always”).

#### Problematic internet use

We used 15-item Generalized Problematic Internet Use Scale (GPIUS2; Caplan, 2010; e.g., “My Internet use has created problems for me in my life”) to measure PIU. Participants expressed their (dis) agreement by responding to Likert type scale varying from 1 (“definitely disagree”) to 5 (“definitely agree”).

#### Emotional intelligence

For measuring EI, a 16-items Wong and Law Emotional Intelligence Scale (WLEIS; Wong and Law, 2002) was used (e.g., “I have a good sense of why I have certain feelings most of the time”; “I always set goals for myself and then try my best to achieve them”). Participants expressed their (dis) agreement on a Likert type scale having endpoints 1 (“definitely disagree”) to 5 (“definitely agree”).

#### Online impulse buying behavior

Online Impulse Buying Behavior was measured using an adaptation of the scale used by Beatty and Ferrell (1998). Multiple studies (e.g., Ahn and Kwon, 2020) have used the adapted version of the same scale to measure online IBB. Respondents marked their response to the 4-items (e.g., “During the visit to electronic commerce website, I saw a number of things I wanted to buy even though they were not on my shopping list.”) on a five-point scale from 1 (“definitely disagree”) to 5 (“definitely agree”).

#### Control variables

To improve the validity and minimize misleading relationships among the study variables, the present study controlled for the following variables: age, gender, experience, and education (Foote and Li-Ping Tang, 2008).

### Method

SPSS (v21.0) was used to extract descriptive statistics, inter-construct correlations, Cronbach’s alpha reliability of the scales, and check the common method bias. Further, AMOS (v21.0) was used for Confirmatory Factor Analysis (CFA), discriminant validity, convergent validity, and the overall model fit of the hypothesized model. Hypotheses H1, H2, and H3 were tested using Multiple regression analysis. The study used PROCESS macro (model 4) for mediation analysis and Model 14 with 5,000 bootstrap samples to conduct the moderated mediation analysis (Preacher and Hayes, 2008).

## Findings and results

### Preliminary findings

Harman’s single-factor test to examine common method bias (CMB). Results exhibit that single-factor accounted for only 32.16% of the variance, suggesting CMB to be less likely Podsakoff et al. (2012). Considering the limitations of Harman’s single-factor method, marker variable method (Lindell and Whitney, 2001) was also deployed. As a marker variable, the current study used a two-item scale of subjective norms specific to physical activity behavior (Courneya et al., 2002; e.g., “Most people who are important to me think I should/should not participate in regular physical activity in the forthcoming month”). The marker variable exhibited insignificant correlation (varies from −0.048 to.001) with all the study variables. Importantly, the marker variable exhibited insignificant correlation with the endogenous variables online IBB (*r* = 0.001, value of *p* = 0.97). Finally, comparison of the parameters—both in the presence and absence of the marker variable—exhibited lower likelihood of CMB among the study variables.

Convergent validity was calculated using average variance extracted (AVE) and Composite Reliability (CR) values. As per the criteria given by Fornell and Larcker (1981), the AVE and CR values should be more than 0.50 and 0.70, respectively. As shown in Table 2, the convergent validity was proved for all the study constructs.

**Table 2:** Composite reliability and convergent validity.

**Construct**	**Items**	**Factor loading ratings**	**Composite reliability**	**AVE**	**MSV**
Mindfulness	12	0.73–0.88	0.84	0.52	0.36
Online IBB	4	0.72–0.93	0.86	0.64	0.41
Problematic Internet Use	15	0.78–0.91	0.82	0.58	0.34
Emotional Intelligence	16	0.71–0.84	0.89	0.62	0.38

Discriminant validity was examined by (a) comparing the square root value of each construct’s AVE with the respective correlation values of the factor construct and (*b*) Maximum Shared Variance (MSV) values, which should be less than AVE values (Fornell and Larcker, 1981). As shown in Table 3, the discriminant validity was established for all the study constructs.

**Table 3:** Descriptive statistics, correlations and discriminant validity.

Construct	Mean	SD	*α*	1	2	3	4
Mindfulness	2.87	0.84	0.96	**0.72**			
Online IBB	2.46	0.97	0.81	−0.15**	**0.80**		
Problematic Internet Use	1.94	0.67	0.93	−0.14***	0.28***	**0.76**	
Emotional Intelligence	2.17	0.85	0.91	0.21*	−0.17***	−0.34*	**0.78**

*p < 0.05; **p < 0.01; ***p < 0.001. Bold values indicate square-root of AVE.

In order to the analyze the validity of the factor structure of the study constructs, we examined the goodness of fit indices. The CFA results are interpreted through various fit indices. The measurement model indices exhibited a good fit (*χ*^2^(196) = 518.24, CMIN/df = 2.64, *p* < 0.001, GFI = 0.89, CFI = 0.97, NFI = 0.93, IFI = 0.97, RMSEA = 0.057), which are in line with the suggestion by Anderson and Gerbing (1988).

### Multiple regression analysis

The results revealed (Table 4) that mindfulness negatively predicted online IBB (*β* = −0.17, *p* < 0.001), supporting H1. Secondly, mindfulness negatively predicted PIU (*β* = −0.11, *p* < 0.001), supporting H2. Thirdly, PIU positively predicted online IBB (*β = 0*.19, *p* < 0.001), supporting H3.

**Table 4:** Results of multiple regression analysis.

Variable	Online IBB (H1)	PIU (H2)	Online IBB (H3)	Online IBB (H4)
*Control variables*				
Age	0.08**	−0.01**	0.07**	0.07**
Gender	−0.01**	0.03*	−0.02**	−0.02*
Experience	−0.06*	−0.07*	0.04**	0.04**
Education	0.05**	0.05**	0.03**	0.03*
*Independent variable*				
Mindfulness	−0.17***	−0.11***	-	−0.13***
*Mediator*				
PIU	–	–	0.33**	–
*R* ^2^	0.18	0.12	0.25	0.29
*F*	14.06***	12.89**	24.76***	13.82***

*p < 0.05; **p < 0.01; ***p < 0.001. Standardized coefficients (IBB, Impulsive Buying Behavior; PIU, Problematic Internet Use). The indirect effect of the predictor (Mindfulness) on the criterion (Online IBB) via the mediator (PIU) is shown when the demographic variables are controlled.

Thus, PIU was considered a potential mediator between mindfulness and online IBB relationship for further examination. The bootstrapping mediation test (using PROCESS Macro Model 4) indicated that the relationship between mindfulness and online IBB was significantly predicted through PIU. The direct effect of mindfulness on online IBB was significant (*β* = −0.13, Standard Error (SE) = 0.04, 95% Confidence Interval (CI) = [−0.03, −0.22]) and the completely standardized indirect effect of mindfulness on online IBB through PIU was also significant (*β*_ab_ = −0.03, SE = 0.02, 95% CI = [−0.07, −0.01]). Thus, H4 was supported. Age and experience have shown a negative impact on PIU, whereas, Gender and Education have shown a positive effect on PIU. Age and education positively impacted online IBB, whereas, Gender and Experience displayed a negative influence on online IBB.

### Test of moderated mediation

**Figure 2:**
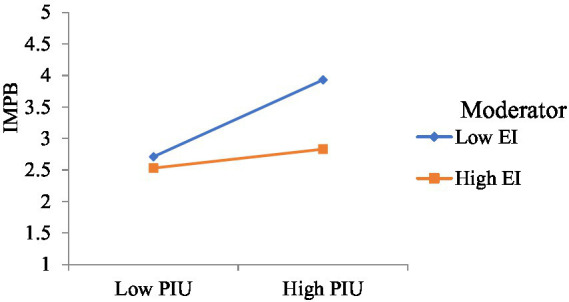
EI moderates between PIU & IBB.

The moderated mediation analysis was performed using the Model 14 of PROCESS Macro (Preacher and Hayes, 2008). The results exhibited that EI acts a negative moderator on the indirect relationship between mindfulness and online IBB through PIU. Results indicate that the negative influence of the cross product between PIU and EI on IBB was significant (*b* = −0.23, *t* = 3.61, p < 0.001). The nature of interaction effect was plotted (Figure 2) using the Aiken and West method (Aiken and West, 1991) of estimating separate equations using one above and below the average of the moderating variable. As hypothesized, the slope of interaction effect shows that the positive relationship between PIU and IBB becomes much weaker with high EI (simple slope = −0.32, *t* = 3.05, *p* < 0.001) than with low EI (simple slope = −0.11, *t* = 2.36, *p* < 0.001). It was observed that across different levels, as none of the levels of CIs included zeros, trait EI significantly moderates the indirect relationship between trait mindfulness and IBB. The index of moderated mediation also supported the finding (*b* = −0.03, 95% CI [−0.04, −0.01]), supporting H5. Thus, EI augmented the strength of indirect effect of mindfulness on online IBB.

## Discussion

Marketing literature is replete with studies exhibiting the psychological and behavioral consequences of impulse buying behavior in the form of post-purchase regret, complaints, negative word-of-mouth messages, and intention to return. Even though “impulsive consumption behaviors are preceded by distinct psychological processes” (Dholakia, 2000, p. 956), these psychological processes have not been understood adequately. This stream of literature is of immense relevance today as mediators and moderators involved in the association between mindfulness and online IBB are expected to provide critical evidence that can enrich our understanding of personal factors as predictors of online IBB, and chart out effective intervention and outreach strategies. In line with the above stated context, the present study attempts to examine the psychological processes that may offer mechanisms to help customers resist impulse buying behavior in online marketplaces.

One of the objectives of the present study was to examine the relationship between trait mindfulness and online IBB, thus reestablishing the importance of mindfulness in facilitating consumer well-being. The present study indicate that mindfulness is negatively associated with online impulse buying behavior, thus supporting H1. This result supports the findings by Dhandra (2020). Theoretically stating, awareness and attentional aspects of mindfulness promote greater emotional regulation, which may help individuals modulate their affective states. A balanced affective state is found to have a negative influence upon IBB (Silvera et al., 2008). Besides, impulsive customers face difficulty resisting the emotional urges related to impulsive online buying. Mindful individuals tend to be more equipped with a deliberative thinking process (think carefully before acting) that allows them to choose their thoughts or behaviors consciously (Kabat-Zinn, 1990) and avoiding reactive approach (Karelaia and Reb, 2015) in decision making situations such as online buying.

The present study results suggest a negative association between mindfulness and PIU, thus supporting H2, which resonates with the extant literature on mindfulness and PIU relationship (e.g., Gámez-Guadix and Calvete, 2016). Mindfulness dismantles the pattern of thoughts (Brown and Ryan, 2003), and reduces automaticity in the thought process and subsequent behavior (Hülsheger et al., 2013). Thus, mindfulness acts as an enabler to present a controlled response to situations, weakening the perpetuation of addictive behavior, including the compulsive urge to use the Internet.

As indicative in Figure 2, people suffering from higher PIU tend to indulge in higher online IBB, thus supporting H3. People under PIU often experience deficient self-regulation, resulting in a relatively depleted conscious self-control (Caplan, 2010). Under such circumstances, these individuals may experience several negative consequences, including impulsive buying behaviors (Vohs and Faber, 2007). Further, because Internet addiction is linked with adverse psychosocial experiences, such as impulsivity, anxiety, and depression (Gecaite-Stonciene et al., 2021), impulsive online buying acts as a behavioral recourse to relieve negative affective experiences for such individuals. Furthermore, individuals high on PIU suffer from a higher preference for online social interaction (POSI) and avoid in-person social interactions. People with high POSI are desirous of deriving interpersonal and social benefits from their online activities (Caplan, 2010). To keep up with the current trends and maintain social status (Amos et al., 2014), excessive digital presence acts as a facilitator for buying impulsively at electronic marketplaces.

Another objective of our study was to examine the novel mediation mechanism of PIU in the relationship between trait mindfulness and online IBB. The study results supported the mediating role of PIU in the association between mindfulness and online IBB, thus supporting H4. To the best of the authors’ knowledge, this study is the first to delve deep into understanding the mediating mechanism of PIU through which mindfulness influences online impulse buying. Mindful non-evaluative awareness and attention advance a greater understanding of the behavioral mechanisms by attending to, acknowledging, and engaging one’s cognition and emotions. Through this, mindful individuals find themselves better equipped to identify the possible antecedents of negative feelings, and are able to maintain a psychological distance from such feelings without getting immersed into it. Such individuals respond constructively and make thoughtful decisions even while attending to uncomfortable affective occurrences (Hayes et al., 2004), thereby reducing their likelihood of indulging in PIU. With higher mindfulness and subsequent lower engagement in PIU, customers may have lower maladaptive cognitions that have the potential to create problems with behavioral impulse control (Davis, 2001), which are significant motivators for IBB (Iyer et al., 2020). Such customers undergo heightened anxiety and have difficulty controlling their affective experiences; they often lose cognitive control and find it more challenging to resist their emotional urges to make impulsive purchases at online marketplaces (Lim et al., 2017). As results suggest, both age and work experience negatively correlate with PIU; the government and social organizations need to devise and develop interventions targeting younger age groups to prevent them from indulging in PIU.

These findings provide evidence of one possible mechanism through which mindfulness impedes online IBB. High-frequency online impulse buying may unsuccessfully act as a diversion technique from day-to-day adverse affective experiences and low self-esteem manifested through PIU. Several mindfulness interventions are found to help enhance the mindfulness level, thus making it a practical means to reduce the tendency to indulge in dysfunctional Internet use. This orientation toward Internet use is expected to maintain the affect balance of customers and make more time available to them to forge face-to-face interpersonal relationships that satisfy the psychological need for relatedness (Li et al., 2016), resulting in reduced negative affect and greater satisfaction with life. Reduced negative feelings are negatively associated with IBB (Verplanken et al., 2005; Silvera et al., 2008).

In line with the third objective of our study, the results establish the moderating role of EI on the association between PIU and online IBB, thus supporting H5. Extant research suggests that the loss of control on emotions (low EI) leads to higher impulsive buying whereas regulating the emotions (high EI) results in lower impulsive buying (e.g., Shams et al., 2021). EI help regulate the impulsivity arising due to PIU. The results (Figure 2) indicate that people with high (low) EI have substantially lower (higher) online IBB at high PIU. The purchase decision process starts with an individual devoting their resources, time, and effort, all of which require cognitive functioning—emotions at work—hence purchase decisions are not immune from emotional interference (Habib and Qayyum, 2018). Once the resources and effort are directed toward purchase by an individual, they are compelled to decide whether to buy or reject the product, and the role of emotions is the most prominent in this phase (Zia, 2019). In order to regulate negative mood states, a customer often uses retail therapy, for example, self-gifting (Rook and Gardner, 1993; Vohs and Faber, 2007), thus falling prey to impulse buying. Proper management and handling of emotions thus becomes imperative to avoid any prompt or impulsive decisions.

## Implications

The study has important implications for theory as well as for organizations.

### Theoretical implications

The study outcomes established the direct and indirect relationship between mindfulness and online impulse buying behavior among individuals. Trait emotional intelligence emerges as a moderator between problematic internet use and online impulsive buying behavior.

The present work establishes that mindful individuals, being aware of the impermanence of the moment can control their impulsive behavior toward online buying. The study also explored the mindfulness-impulse buying behavior relationship through the indirect mechanism of PIU and established that in the presence of greater mindfulness, an individual’s PIU will reduce significantly, thus reducing their intention to splurge through online impulse buying. In addition, higher emotional intelligence helps individuals respond to the cues in the system in more desirable ways.

While studies explaining the causes and consequences of online impulsive buying behavior are present in the extant marketing literature, empirical studies demonstrating the intrapsychic processes through which online IBB could be controlled are scant. The study outcomes attempt to fill this critical gap, which has multiple implications at individual, societal, and organizational levels. Under the influence of carefully propagated consumerist messages and a relative absence of individual strength, online impulsive buying creates an illusion of short-term sense of accomplishment and makes customers sacrifice their long-term financial well-being (Fenton-O'Creevy et al., 2018). The outcomes empirically demonstrate mindfulness is negatively associated with online IBB as well as with PIU. Thus, a mindful customer may find it easier to regulate their impulsive desire to splurge at online marketplaces by virtue of enhanced mindfulness and subsequent reduced PIU. Besides, by demonstrating the moderating role of trait EI on the relationship between PIU and online IBB, we also extended the theoretical framework of self-regulation by explaining the role of psychological variables in guiding the customers to refrain from mindless as well as impulsive splurging in the online realm.

This study presents two novel theoretical contributions. First, the study establishes the mediating role of PIU in the relationship between mindfulness and online IBB. Second, the role of EI as the moderating factor between PIU and online IBB is another salient finding. To the best of the authors’ knowledge, these relationships are accounted for the first time in academic literature. In addition, the findings are critical to explain how mindfulness impacts numerous other factors, particularly those associated with sudden and unplanned online IBB. The results are important for describing the ways that can help people to evaluate and control their sudden urge to indulge in online IBB.

### Implications for organizations

Online marketers often use situational antecedents such as high-quality personalized product recommendations, that optimize a customer’s search for products and further, fuels online IBB (e.g., Ampadu et al., 2022). Apart from adverse financial outcomes that lead to detrimental effects on consumer well-being (Fenton-O'Creevy et al., 2018), e-commerce firms too sometimes suffer from customers’ high impulse buying behavior, leading to complaints, and costs associated with product return and reverse logistics (Lim et al., 2017). Unlike the offline brick-and-mortar shopping environment, the return of physical goods incurs additional handling costs in online marketplaces. Such situations can adversely affect the economic objectives of online sellers and electronic commerce businesses.

As discussed, customers with a higher propensity to respond to impulse buying often express regret, have a higher intention to return goods, and describe complaining behavior (Zeelenberg et al., 2000). It is noteworthy that complaining behavior is usually preceded by regret caused by impulsive buying. The salient effect of mindfulness in helping individuals exhibit the behavior they will not regret later is well established (Bristow, 2019). Being mindfully aware can contain the adverse affective experiences in ruminative tendencies, which often leads to regret (Nolen-Hoeksema et al., 2008).

The study finds that mindfulness is negatively associated with online IBB as well as with PIU. PIU is associated with multiple detrimental psychosocial outcomes and adverse emotional experiences (Caplan, 2002; Sinha et al., 2021a). Since the essential features of e-commerce websites may play a significant role in triggering emotions to stimulate the impulse buying behavior (Park et al., 2012), e-commerce firms may look at ways to institutionalize the embedded mechanisms (e.g., pop-up messages) within their interactive web portal framework to boost mindfulness of the customers which, in turn, reduces the dysfunctional usage of the Internet in the form of PIU Such external interventions are effective in augmenting customers’ awareness level (Monaghan, 2008), allowing them to make informed purchase decisions. Supporting this, studies have empirically established that, unlike other trait level variables, people can be trained to be more mindful across various life stages and age groups through physical and online or app-based mindfulness techniques (Brown and Ryan, 2003; Chittaro and Vianello, 2016). Thus, online marketers may guide the customers in reaping the benefits of mindfulness directly and through reduced PIU in evaluating their online IBB. Such external interventions may make it easier for customers to regulate their impulsive purchase intentions at the online marketplace.

The study outcomes reveal that EI, which is understood as the ability to identify and utilize emotions to prevent extreme emotional experiences, moderates the relationship between PIU and online IBB. Acting as an automatic regulatory resource (Gooty et al., 2014), the presence of higher EI assists individuals in regulating their unplanned and sudden urge to buy at the online marketplace. In a meta-analytic study, Iyer et al. (2020) identified ‘marketing drivers’ as one of the three factors responsible for IBB. In the presence of low levels of EI, marketing instruments and activities trigger the desire to indulge in impulsive purchase activities (Mattila and Wirtz, 2001). The present study finds that high levels of EI, acting as a buffer, weakens the strength of the relationship between PIU and online IBB. Thus, individuals with high EI levels are less prone to be affected by the adverse effects of PIU on their online IBB.

Developing an emotional bond between customers and the company is not easy in the online context (Barari et al., 2021). Embedding such an external mechanism within the web framework of electronic commerce organizations may benefit online marketers in regulating the unplanned purchase process—while restricting completely uninhibited buying behavior that leads to regret, and the subsequent intention to return products—and have vital implications for customer relationship management (Lim et al., 2017). Researchers are of the opinion that “ultimately, marketers must choose between making an immediate sale that might produce consumer dissatisfaction and exhibiting concern for the consumer to encourage future patronage” (Iyer et al., 2020, p. 400).

### Social implications

Consumers spend $5,400 annually on impulse purchases, adding to their financial woes (O’Brien, 2018). Such impulse buying behavior is associated with several adverse consequences, such as financial difficulties (Fenton-O'Creevy et al., 2018) and lower financial well-being (Nanda and Banerjee, 2021). With the savings rate at a record low, financial well-being is found to influence the overall well-being of individuals negatively and has attracted considerable attention in academic research (Netemeyer et al., 2018; Sinha et al., 2021b) and public policy (e.g., OECD, 2020). Many people facing diminished financial well-being create societal problems, leading to adverse welfare effects for today and in the future (Brüggen et al., 2017). The study outcomes demonstrate that mindfulness relates negatively to online IBB directly and through PIU, thus elucidating possible mechanisms through which individuals’ online IBB can be contained, potentially influencing the individual financial well-being and overall well-being positively.

## Limitations and areas for future research

The present study has the following limitations. First, the participants were more educated than the population at large, and from India. Future studies may look at the relationship between mindfulness and online IBB relationships in samples from different cultural contexts (*viz.* collectivist vs. individualist) to gather novel insights and augment the generalizability of the study findings. Second, though we looked at ways to address the issue of common method bias, the findings should be interpreted with the usual cautions associated with cross-sectional self-reported data. Further studies may look at the mindfulness intervention design to validate the findings of this research. Third, though the present study factored in the role of demographic variables while examining the direct and indirect relationships, there may be other psychological processes that might affect online IBB. These factors such as materialism, self-esteem should be controlled in future studies. Fourth, considering the wide variety of factors as the antecedents of PIU and subsequent online IBB, future research should look at the impact of other psychological factors and processes. Lastly, future studies may focus on validating the proposed conceptual model for more sector specific outcomes in both emerging and developed economic contexts.

## Data availability statement

The raw data supporting the conclusions of this article will be made available by the authors, without undue reservation.

## Ethics statement

The studies involving human participants were reviewed and approved by Ethics Committee of Chitkara University, Punjab, India. The patients/participants provided their written informed consent to participate in this study.

## Author contributions

NV and NS conceived of the presented idea and developed the theory. NS, AT, and SM conducted the analysis. NV has verified the analysis. All authors contributed to the article and approved the submitted version.

## Conflict of interest

The authors declare that the research was conducted in the absence of any commercial or financial relationships that could be construed as a potential conflict of interest.

## Publisher’s note

All claims expressed in this article are solely those of the authors and do not necessarily represent those of their affiliated organizations, or those of the publisher, the editors and the reviewers. Any product that may be evaluated in this article, or claim that may be made by its manufacturer, is not guaranteed or endorsed by the publisher.
